# One step beyond the lab and clinic: “walking the dementia conversation”

**DOI:** 10.3389/fpubh.2023.1284692

**Published:** 2023-12-08

**Authors:** Jonathan Adrián Zegarra-Valdivia, Fernando Aguzzoli-Peres, Alex Kornhuber, Faheem Arshad, Carmen Noelia Paredes-Manrique

**Affiliations:** ^1^Global Brain Health Institute – University of California, San Francisco, San Francisco, CA, United States; ^2^Trinity College Dublin, Dublin, Ireland; ^3^Faculty of Health Sciences, Universidad Señor de Sipán, Chiclayo, Peru; ^4^National Institute of Mental Health and Neurosciences (NIMHANS), Bangalore, India; ^5^Universidad Tecnológica del Perú, Lima, Peru

**Keywords:** patients, dementia, empathy, openness, research participation

## Abstract

Millions of dollars have been lost in dementia research over the last 30 years owing to unsuccessful clinical trials aimed at finding an effective treatment for the condition. Although two promising drugs have been identified, the research effort is insufficient. The dehumanization of patients and the pressure to publish have led to a decline in the quality and usefulness of scientific research. One way to tackle these problems is establishing close contact with those who put their faith in researchers. Fine-tuning the participation of patients with dementia and caregivers in research design and improving their connection and communication with researchers could positively contribute to enhancing the perspectives and designing strategies for scientists in order to generate a new and enriching vision. The Walking the Talk for Dementia event showed that people can still live with dementia despite their condition. Approximately 300 people participated in the all-week “Santiago's Camino” symposium. People living with dementia, caregivers, healthcare professionals, activists, clinicians, and researchers participated in this event. The “Walking the Talk for Dementia” (WTD) event vividly demonstrated a strong commitment to upholding Global Brain Health Institute's (GBHI) core values of Authenticity, Fairness, Openness, Respect, Courage, and Empathy (A FORCE) to advance equity in brain health. These values provide clear guidance for their advocacy initiatives aimed at mitigating the global impact of dementia. Research and development are essential across scientific fields, especially in clinical contexts where involving patients and caregivers is critical. The WTD initiative exemplifies this aspect by bringing together researchers, caregivers, and dementia patients on the Camino de Santiago in Spain.

## Introduction

Dementia is an advanced state of cognitive and functional deterioration often associated with advanced age (1), but it can also affect a large, relatively young population under 60 years (2). Although some types of dementia, such as Alzheimer's, Parkinson's, or others, are well-known, there are many causes of dementia and even intermediate phases between the condition and cognitive and functional normality (3). It is known that the prevalence of dementia is increasing worldwide, and according to the World Health Organization (4), more than 55 million people currently suffer from it, with an increase of 10 million cases annually. By 2050, an additional 152 million people are estimated to be affected by dementia (5). Out of those affected, nearly 60%−70% of cases are associated with Alzheimer's disease (4).

Although a vast amount of preclinical research has been carried out to elucidate causes, mechanisms, and therapeutic approaches, and hundreds of clinical trials have been carried out in search of specific treatments, few results have been genuinely fruitful but have been abandoned (6). There are currently two drugs that the scientific community and the US Food and Drug Administration (FDA) have paid attention to for the treatment of Alzheimer's dementia: lecanemab (7, 8) and aducanumab (9–11). In this context of initial FDA-approved treatments, and despite controversies (9), several questions arise for the researchers. To what extent is researchers' knowledge of patients established? To what extent does the medical professional know the individuals being treated? What are patients' feedback regarding the study conducted and the allocation of funding and resources?

## Walking the talk for dementia conversation

**Figure 1:**
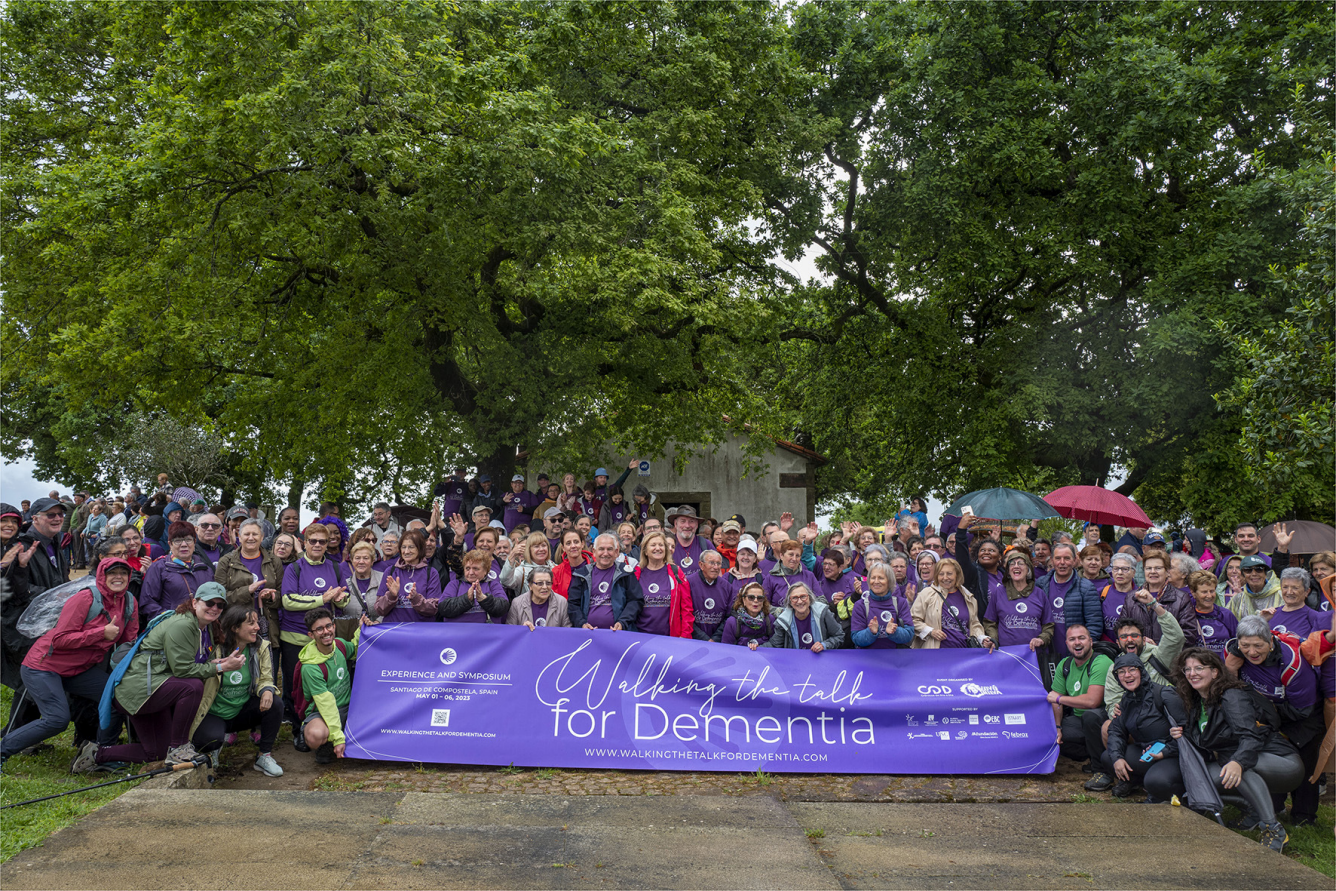
The “Walking the Talk for Dementia” event, held on April 2023 in Santiago de Compostela, was a 5K walk organized by the Spanish Association of Family Members of People with Dementia (AFA) and the Spanish Association of Alzheimer's (AE Alzheimer), Global Brain Health Institute (GBHI), and other institutions. Drawing over 300 participants, the WTD aimed to raise funds for dementia research and care. The event featured an information fair on dementia, providing knowledge about the disease and ways to support individuals with dementia and their families.

We recently had the opportunity to participate in an event called “Walking the Talk for Dementia.” In this event, participants worldwide were invited to walk the Camino de Santiago, with the aim of raising funds for dementia research and care, and the event managed to attract more than 300 participants. The event featured an information fair on dementia, providing knowledge about the disease and ways to support individuals with dementia and their families. This pilgrimage route spanned several paths, leading to the city of Santiago de Compostela, located in the northwest of Spain. Pilgrims travel to this city in search of the tomb of the Apostle Santiago. In this event, different world-class researchers in dementia, including Spanish researchers, caregivers, and even patients living with dementia were invited to follow this path over 4 days (see Figure 1).

## Methodology

The necessary logistics for conducting the WTD (ambulances, paramedics, caregivers, transport buses, and other necessary equipment) was arranged by two Brazilian organizations, Instituto Vovó Nilva and Associação Crônicos do Dia a Dia (CDD), a Brazilian NGO focused on chronic patients. CDD predominantly funded the project with the support and collaboration of the Alzheimer's Disease International (12), Global Brain Health Institute (13), Atlantic Fellows, and others. This event culminated in a 2-day symposium, where the experiences of various patients and researchers were presented.

This event saw 300 participants (from 25 countries, spanning five continents) walking through the “Camino de Santiago” for 4 days and attending the final 2-day symposium. Regarding patients with dementia, 10 of them participated through the week from six countries with diverse cultural backgrounds (United States, Ireland, England, Singapore, Spain, and Namibia). Otherwise, a majority of dementia patients participated for only 1, 2, or 3 days.

The objective of the event was to establish an atmosphere conducive to open and non-judgmental dialogues among individuals living with dementia, caregivers, healthcare professionals, and policymakers. Those living with dementia and their caregivers, after providing consent, openly shared their individual experiences with the intent to raise awareness. No formal clinical assessment, neuroimaging, or treatment (specific clinical data) was shared during the symposium by the organizers. A signed speaker release form for the recording was obtained and the event was then podcasted.

### A different staging

Contrary to the formality of symposiums and scientific events, WTD was an event for all those involved in dementia care. The openness in communication, the dialogue, the straightforward explanations, and the interactions were a rare occurrence but provided rich experiences for the participants, among whom patients with Alzheimer's dementia and those with lewy body dementia (14) were present. The 2-day symposium began with presentations by some researchers, moderated by other attendees, who often turned out to be patients invited by the organizers. The presentations also included exhibiting patients, during which the attendees were seated on round tables so that everyone's voice was heard. This event demonstrated a practical way of fostering patient and public involvement (PPI), a growing methodology for inviting patients and the public to participate in research, its implementation, planning, and active participation (15). Although the traditional way of implementing a PPI goes hand in hand with committees organized by the investigators, the staging was practically driven by patients and their caregivers. As GBHI fellows, we aspire to improve brain health and reduce the global impact of dementia by reaching out to the local communities and worldwide networks. Our core values at GBHI are symbolized by the acronym “A FORCE,” which signifies Authenticity, Fairness, Openness, Respect, Courage, and Empathy. Therefore, we embrace these values to ensure brain health equity. These values guide us as we advocate for reducing the global impact of dementia. By adopting these value-driven approaches to brain health, science, arts, humanities, and advocacy, we can drive change for millions of people with dementia. “Walking the Talk for Dementia” as a novel event exemplified this transformation and displayed our core values.

### Authenticity

The event was initially conceived by a Brazilian individual, Fernando, who at present is deeply immersed in dementia research due to his professional background. However, it all began when he decided to drop his philosophy studies and dedicate himself to caring for his grandmother with Alzheimer's for over 6 years. Fernando and his partners did not want to create something for people with dementia but rather to work with people with dementia, which is why Laureen Waters, a person living with Alzheimer's in South Carolina, United States, was invited to join the board and take part in organizing the event. Her presence ensured that the perspective of individuals with dementia would be represented and influence important decisions regarding the WTD.

### Fairness

In PPI committees, participants are often invited to help in different phases of the investigation, such as selecting instruments that the investigator will need to assess other patients with the same disease, and that will serve to address problems with which they live on a day-to-day basis. The patients' approach can generate a completely new vision with a different and enriching interpretation for researchers (15).

### Openness

Experiencing dementia within the closest family circle is a profoundly transformative journey filled with dramatic life changes and ever-changing emotions. It is only natural that this experience shapes our perception of people with dementia, extending beyond our immediate family. Fortunately, not all professionals dedicated to developing diagnostic alternatives, treatments, and care for people with dementia have had the opportunity to personally experience it within their own families. Therefore, it is of the utmost importance to foster experiences that can, in some way, recreate these relationships, forging strong bonds between both sides of this complementary universe formed by the diagnosed individuals and those seeking solutions to facilitate or improve their lives within this context.

#### Courage

Regarding this practice, we consider that the primary use is the humanization of the patient, contrary to dehumanization in medicine and other professions related to patient care (16). Dehumanization, which in many countries is increased by the protocol or policies of the ministries of health that impose a limit of patients added to the administrative burden that they may have, cut the time of patient care, and thereby limit the listening to the patient part. In WTD, being able to talk informally with patients, the fact that they go on stage and present their experiences and their needs or that caregivers talk about the importance of the perception of signs, which is different for caregivers and also for people who take care of these caregivers showed the importance of returning to a fundamental question in bioethics for researchers: For whom do we do what we do?

#### Respect

Taking a broader perspective, and, in this section, from the point of view of clinicians rather than researchers, the interaction with individuals diagnosed with dementia is part of their daily lives but in a different context than what was experienced during the WTD. In clinical roles, we are accustomed to evaluating capabilities and constantly searching for markers that indicate when it may be time for individuals to stop driving, traveling, or taking risks. However, the WTD provided a unique and transformative experience far removed from our usual clinical practice. It allowed us the space to witness the element of surprise and be genuinely impressed by the determination of individuals living with lewy body dementia. Many of them, who were once limited to walking just a block and doubted by their doctors, defied expectations by walking 5 km or more each day.

#### Empathy

We believe that the WTD has introduced an innovative approach to scientific conferences, emphasizing genuine, deep, and enriching exchanges among professionals, researchers, and individuals living with dementia. Over 4 days, walking 10 km daily, this experience creates a unique environment where personal and professional experiences, expertise, knowledge, vulnerability, emotions, and expectations can be shared. Through the choice of walking companions and engaging in meaningful conversations about dementia, participants have the opportunity to step out of their laboratories and “walk in each other's shoes,” resulting in a profound and impactful transformation.

When Kevin Quaid, living with lewy body dementia and Parkinson's dementia, from Ireland, was invited by Fernando to join the WTD, his initial reaction was, “Do you think I can? Do you think I'm capable?” Fernando responded, “I think enough people are telling you what you can or cannot do, should or should not do. So, I want to hear from you. Do you believe you can? And more importantly, do you want, dude?” Kevin replied that it would be a dream come true but not without shedding a few tears. The overwhelming emotion was not solely about the imminent journey to Spain or the adventure itself, but the first profound impact for Kevin realized that someone genuinely valued his voice and opinion himself.

We agree that the basic answer would be by and for the patients. However, in the current research context, researchers often develop their work in a rush and under pressure to publish because of incentives and recognition, often leaving scientific quality aside (17). It is where the researcher's bioethical principles are lost.

The interaction with patients who live with the disease day-to-day allowed them to develop empathy; the shared environment and the stories sharpened assertiveness, and their coexistence promoted understanding. We believe that these aspects were central to this event, which united all those involved in dementia and glorified new ties.

In this manner, the PPI shows its importance and use in research; however, it has not been implemented in several Latin American countries; on the contrary, it entails using resources that are often unavailable (18). In addition to this fact, the dehumanization of patients and the immediate search for the publication of scientific texts (16, 17), without taking care of the quality, is generating excessive publications without scientific impact and, above all, without clinical utility. Many problems in bioethics, research, development, innovation, and clinical, social, and technological usefulness may be in decline because those who live with dementia or those who care for them are not being considered. After all, the researchers are dehumanizing those suffering from the disease or for not listening to those who know the most about the condition they live with.

### The stigma problem

The problem of stigma in dementia is that patients and relatives can perceive the social, family, and work effects of a diagnosis (19). However, the lack of knowledge in society about these disorders often erroneously stigmatizes patients in relation to the factors that they have been traditionally associated with.

In the case of dementia, it is believed that patients are completely unaware of the disease or of the cognitive or functional deficit they suffer from, or on the contrary, these deficits are normalized with comments such as: “It's part of old age,” “It's/you are old,” and “It is normal for you to forget,” among others. It is believed that aging is necessarily related to a loss of cognitive processes, normalizing the presence of signs and symptoms associated with various pathologies associated with dementia or age [Alzheimer's disease (20), frontotemporal dementia (21), vascular dementia (22), and others] being, on the contrary, characteristics that appear at the beginning of these pathologies. By normalizing them, we only accentuate the progression of these disorders by avoiding their early diagnosis and treatment; thus, the stigma in dementia and other diseases often promotes ignorance and fear while exacerbating the chronicity and accentuation of deficits.

Thus, researchers must consider their work's ethical implications, behavior, and disclosure to society, considering their work's clinical utility and impact. A prevailing need is to be clear about national and international regulations when planning their investigations, the consideration and involvement of patients and the public in the different phases of the research, and adherence to clear ethical rules.

The confidentiality and privacy granted to the patient, the simple communication with them, or the review that the protocols are irrefutably followed is not enough for adequate ethics, but, on the contrary, it is necessary to add the search for the scientific, social, and clinical impact of our work, by and for patients and their families, and it should be the common goal to develop replicable, verifiable, and helpful research. In this way, we not only avoid potential damage to the study (for example, in its verifiability) or to the patients but, on the contrary, in relevant emotional damage to the family, the patient, society, and the scientific community.

It is also necessary for researchers to consider the possible social and cultural implications of their research, its credibility with society, and the educational impact on the training of new researchers; this is particularly relevant since university students see research as a complicated, inaccessible, complex topic that adds to university difficulties and impairs their development (23, 24).

#### The road does not end in Santiago. It begins there

Implementing a novel methodology for the participation of patients, caregivers, and researchers left memorable experiences. It reaffirmed the fundamental ethical aspects to continue to be focused on patients and their families, such as assertive communication by clinicians, empathy by scientists, the development that integrates patients and caregivers, and above all, the redirection of funds to better-designed jobs involving all participants in an inclusive, diverse, and equitable way.

The failure of many clinical trials has been due to several factors. We wish to emphasize the inadequate characterization of patients with dementia, such as Alzheimer's, as there are multiple types of dementia. This is due to the syndromes generated, whether typical or atypical (25), brain regions affected, and unknown interaction of factors in these disorders, and could have been avoided in many instances with the formal involvement of caregivers and patients in the investigations through the PPI, in addition to supporting personnel who report information and researchers who collaborate in the design and identification (15).

When you bring together such contrasting and distinct cultures in the same environment, representing all continents but fostering a conducive space for exchanging experiences, you become acutely aware of the research world's entrenched racial and social inequities. While some communities do not feel adequately represented by research groups advancing studies that fail to involve their members inclusively, the campaigns and well-known figures participating in the PPI panels are typically white individuals from developed countries. Low- and middle-income countries (LMICs) face significant challenges in finding appropriate representation and embodiment of dementia experiences within their nations. These challenges stem from numerous intertwined problems, including stigma, delayed diagnoses, and low scientific literacy. These aspects feed into one another, preventing the voices of these communities from being heard and their needs from being effectively addressed.

All the reflections heard at the symposium allowed a genuine renewal of the interests of researchers and renewed the expectations of patients and their families, but above all, it allowed them to talk about issues that are often not talked about, to listen to those who are not heard too, and also encouraged them to think from divergent perspectives, all with a common goal, to face dementia.

## Conclusion

Research, innovation, and development are necessary for all branches of science. However, in those that involve the clinical context, it is even more critical to apply the patients themselves and their caregivers, learn about their perspectives in research, and conduct research in ways that humanize the patients. However, we must consider how science is communicated and how learning and sharing transfer spaces have been built. This step is beneficial and enriching. The experience of “Walking the Talk for Dementia,” an event that brought together researchers, caregivers, and patients with dementia and allowed them to walk together on the Camino de Santiago in Spain, has opened the possibilities for various purposes and perspectives. In this manner, we would like to highlight that the launch of this event was a practical way of proposing “patient and public involvement” (PPI), a growing methodology of inviting patients and the public to participate in research and implementation of the design of clinical trials. This would ensure that the voices of caregivers and patients are heard, and an enriching vision can be achieved for researchers.

The “Walking the Talk for Dementia” initiative served as a platform to raise awareness and funds for dementia research and care, bringing together patients, attendees, participants, clinicians, researchers, caregivers, and family members. Their dedication paves the way for impactful collaborations in the future, contributing to improving the lives of individuals affected by dementia and their families.

## Limitations

Within the parameters of the event, several limitations were discerned. First, the event's funding was limited, which would limit the number of participants, precedence, and location. The program's relatively brief duration restricted its capacity to encompass the full spectrum of experiences among individuals living with dementia and their care partners on a global scale. While we secured some funding to diversify participant representation, the event primarily relied on voluntary participation contributions contingent upon the availability of participants' time and resources. The reliance on voluntarism may have constrained both the event's outreach and its potential impact, potentially compromising the richness of participant diversity and experiences.

The event was organized primarily by Brazilians and Spaniards and took place in Spain. Still, in order for it to be a global event and bring multiple perspectives, English was adopted as the official language for the event. This decision limited our reach significantly, especially considering that individuals from disadvantaged social backgrounds in Latin America were unable to attend. Finding ways to overcome this challenge should be further explored in future events, ensuring more diversity and inclusion; for example, the incorporation of online language translation services, although beneficial, necessitated additional funding.

It is noteworthy that, with regard to data collection, the event organizers did not acquire any formal or clinical data from participants. In future events, the inclusion of qualitative methodologies and orientation toward focus groups for initial public and patient involvement (PPI) activities could prove advantageous.

By addressing these limitations in upcoming dementia-related events, there is a significant potential to augment their effectiveness in the ongoing battle against these debilitating diseases.

## Data availability statement

The raw data supporting the conclusions of this article will be made available by the authors, without undue reservation.

## Ethics statement

Written informed consent was obtained from the individual(s) for the publication of any potentially identifiable images or data included in this article.

## Author contributions

JZ-V: Conceptualization, Funding acquisition, Writing – original draft, Writing – review & editing, Validation. FA-P: Conceptualization, Writing – original draft, Writing – review & editing. AK: Writing – original draft, Writing – review & editing. FA: Writing – original draft, Writing – review & editing. CP-M: Writing – original draft, Writing – review & editing.
